# Ensemble-Based Computational Approach Discriminates Functional Activity of p53 Cancer and Rescue Mutants

**DOI:** 10.1371/journal.pcbi.1002238

**Published:** 2011-10-20

**Authors:** Özlem Demir, Roberta Baronio, Faezeh Salehi, Christopher D. Wassman, Linda Hall, G. Wesley Hatfield, Richard Chamberlin, Peter Kaiser, Richard H. Lathrop, Rommie E. Amaro

**Affiliations:** 1 Department of Pharmaceutical Sciences, University of California, Irvine, California, United States of America; 2 Department of Biological Chemistry, University of California, Irvine, California, United States of America; 3 Institute for Genomics and Bioinformatics, University of California, Irvine, California, United States of America; 4 Department of Computer Science, University of California, Irvine, California, United States of America; 5 Department of Chemistry, University of California, Irvine, California, United States of America; University of California San Diego, United States of America

## Abstract

The tumor suppressor protein p53 can lose its function upon single-point missense mutations in the core DNA-binding domain (“cancer mutants”). Activity can be restored by second-site suppressor mutations (“rescue mutants”). This paper relates the functional activity of p53 cancer and rescue mutants to their overall molecular dynamics (MD), without focusing on local structural details. A novel global measure of protein flexibility for the p53 core DNA-binding domain, the number of clusters at a certain RMSD cutoff, was computed by clustering over 0.7 µs of explicitly solvated all-atom MD simulations. For wild-type p53 and a sample of p53 cancer or rescue mutants, the number of clusters was a good predictor of *in vivo* p53 functional activity in cell-based assays. This number-of-clusters (NOC) metric was strongly correlated (r^2^ = 0.77) with reported values of experimentally measured ΔΔG protein thermodynamic stability. Interpreting the number of clusters as a measure of protein flexibility: (i) p53 cancer mutants were more flexible than wild-type protein, (ii) second-site rescue mutations decreased the flexibility of cancer mutants, and (iii) negative controls of non-rescue second-site mutants did not. This new method reflects the overall stability of the p53 core domain and can discriminate which second-site mutations restore activity to p53 cancer mutants.

## Author Summary

p53 is a tumor suppressor protein that controls a central apoptotic pathway (programmed cell death). Thus, it is the most-mutated gene in human cancers. Due to the marginal stability of p53, a single mutation can abolish p53 function (“cancer mutants”), while a second mutation (or several) can restore it (“rescue mutants”). Restoring p53 function is a promising therapeutic goal that has been strongly supported by recent experimental results on mice. Understanding of the effects of p53 cancer and rescue mutations would be helpful for designing drugs that are able to achieve the same goal. The challenge is that cancer and rescue mutations are distributed widely in the protein, and experimental testing of all possible combinations of mutations is not feasible. This paper describes a simple computational metric that reflects the overall stability of the p53 core domain and can discriminate which second-site mutations restore activity to p53 cancer mutants.

## Introduction

**Figure 1:**
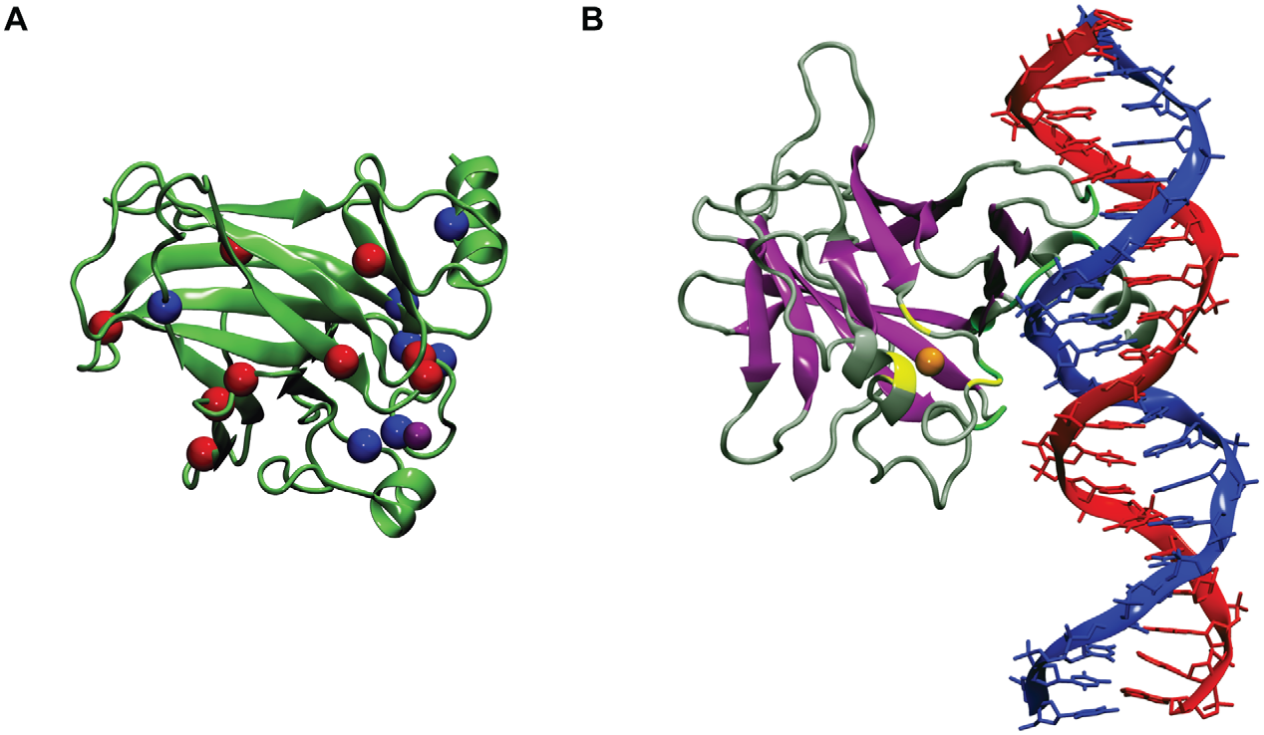
p53 DNA-binding core domain. A) p53 DNA-binding domain mutations studied in this work. The zinc ion, destabilizing cancer mutations and stabilizing rescue mutations focused are depicted in purple, blue and red spheres, respectively. B) Different types of mutations in the p53 DNA-binding core domain. β-sheet residues, zinc-binding residues and DNA contact residues are depicted in purple, yellow and green, respectively. The zinc ion is depicted as an orange sphere.

The tumor suppressor protein p53 is a transcription factor that plays a major role in preventing cancer initiation and progression. Cellular stress conditions such as hypoxia or DNA damage activate p53, which induces cell cycle arrest, DNA repair, senescence, or apoptosis [1], [2], [3]. In most, if not all, human cancers, the p53 apoptosis pathway is inactivated, and p53 itself is mutated in about half of all human cancers. About three-quarters of tumors with mutant p53 express full-length p53 with single missense mutations in the p53 DNA-binding core domain. These mutations may cause partial or global protein destabilization, loss of zinc coordination, or disruption of DNA contacts, and thus inactivate the tumor suppressor function of p53 (www-p53.iarc.fr) [4]. These missense mutations (“cancer mutations” or “oncogenic mutations”) are widely distributed throughout the core domain (Figure 1). They have been classified based on their physical location within the protein: (i) DNA-contact mutants (e.g., R248Q, R273H), (ii) structural mutants in the DNA binding surface (e.g., R175H, G245S, R249S, R282W), (iii) β-sandwich mutants (e.g., Y220C), and (iv) zinc-binding domain mutants (e.g., C242S, R175H).

Pharmacological rescue of p53 function in cancer tissues is an attractive therapeutic target [5]. Recently, two independent studies on transgenic mice demonstrated that restoration of p53 activity enables tumor regression *in vivo* [6], [7]. p53 reactivation is especially promising in regression of advanced stage cancers [8], [9]. The p53 function of some oncogenic mutants has been rescued *in vivo* by a handful of small molecules [10], [11], [12], [13], [14] as well as by second-site suppressor (“cancer rescue”) mutations [15], [16], [17], [18]. The second-site mutations provide easily-studied cases of p53 cancer rescue.

The effect of oncogenic and rescue mutations in p53 has been of great interest. Many detailed structural studies have been pursued, including X-ray crystal structures of individual oncogenic and rescue mutants of p53 [19], [20], [21]. The loss or gain of hydrogen-bonding interactions, salt bridges and other minute stabilizing or destabilizing effects upon different missense mutations have been investigated to develop a more complete understanding of the inactivation mechanisms by the oncogenic missense mutations and, correspondingly, the mechanisms by which restoration of activity for rescue mutations occur [22], [23]. At 310 K, wild-type p53 is estimated to be only 3.0 kcal/mol more stable than the denatured state [23], and thus missense mutations can easily shift the delicate balance of p53 stability.

The present study quantifies the effect of oncogenic and rescue mutations on the overall dynamics of p53 without focusing on local structural details. The core DNA-binding domain of p53 was used, as it dictates the stability of the overall protein [22]. The overall protein flexibility of the p53 DNA-binding domain for the wild-type, cancer mutants, rescue mutants and non-rescue mutants was compared in explicitly-solvated all-atom molecular dynamics (MD) trajectories, which are well suited to investigate the local conformational space sampled by each particular mutant. A single discriminating metric, the measure of flexibility of p53 in terms of the number of clusters obtained at a certain RMSD cutoff, was able to predict the functional activity of various mutant p53 proteins.

## Methods

### System preparation

The coordinates for the starting structure were obtained from the wild-type p53 coordinates of chain B in pdbID 1TSR [4]. Each mutant system was prepared from this structure by rebuilding the mutated side chain(s) with the AMBER suite [24]. Crystallographic waters were retained. Histidine, asparagine and glutamine side chains which were mis-fit during structure characterization were determined and flipped by 180° using the Molprobity web server [25]. Histidine protonation states were determined using the Whatif Web Interface [26] and manually verified. Zinc coordination residues (Cys176, Cys238, Cys242 and His179) were modeled following the cationic dummy atom method of Pang *et al* [27]. Missing atoms and hydrogens were added using the Leap module of Amber10 [24]. Each system was solvated in a TIP3P [28] water box. The buffer between the protein and the periodic boundary was not closer than 8 Å in any direction. The wild-type p53 system has a charge of +1. Chloride ions were added as needed to neutralize the different mutant systems studied. The topology and coordinate files of the systems were constructed using Amber FF99SB force field [29]. The final wild-type p53 system consisted of 27,264 atoms.

### MD simulations

Each system was first relaxed by 36,000 steps of minimization and a standard relaxation procedure using restrained MD. In the first 2,000 steps of minimization only the hydrogen atoms were relaxed, leaving all other atoms fixed. In the second 2,000 steps, all water atoms and ions were minimized in addition to the hydrogen atoms. In the third 2,000 steps, zinc-coordinating residues Cys176, Cys238, Cys242 and His179 as well as all hydrogens, water atoms, and ions were minimized. In the following 10,000 steps, all atoms were minimized except backbone atoms, which were held fixed. In the last 20,000 steps, the entire system was minimized. Following the minimizations, restrained MD simulation at 310 K was carried out for 1 nanosecond to prevent structural artifacts from introducing kinetic energy into the system. For this purpose, positional restraints for the heavy atoms of the protein backbone were gradually decreased from 4.0 to 1.0 kcal/(mol * Å^2^) in four consecutive 250-picosecond-long MD simulations.

Thereafter, unrestrained MD was performed in explicit solvent for 30 nanoseconds at 310 K using a time step of 1 femtosecond. Temperature was maintained constant at 310 K by Langevin dynamics with a collision frequency of 5 ps^−1^, and pressure was maintained at 1 atm by the Nose Hoover-Langevin piston method [30], [31] using period and decay times of 100 and 50 femtoseconds, respectively. Long-range electrostatics was treated by the Particle Mesh Ewald method [32] and a nonbonded cutoff of 10 Å was used. The interatomic distances within the water molecules were fixed using the SHAKE algorithm [33], [34]. A multiple-time step algorithm was employed, in which bonded interactions were computed at every time step, short-range non-bonded interactions were computed at every second time step, and full electrostatics was computed at every fourth time step.

All minimizations and MD simulations were performed using NAMD2.7 [35] on the Teragrid Ranger cluster. The simulations scaled as 0.10 days per nanosecond using 64 processors. Root-mean-square-deviation (RMSD) traces over the course of the MD trajectories are depicted in Figure S1.

### Mutants considered

We considered all four structural classes of p53 mutants: (i) DNA-contact mutants, (ii) structural mutants in the DNA binding surface, (iii) β-sandwich mutants, and (iv) zinc-binding domain mutants. We did not attempt to characterize any direct zinc-binding residue mutations (e.g., C242S), as rigorous parameterization of the partial charges on the metal ion and coordinating groups would be required for proper treatment of any mutations in this area.

Mutants simulated included the wild-type p53, the six most-frequent cancer mutants (R175H, G245S, R248Q, R249S, R273H, R282W), cancer mutant Y220C for which some stabilization (although not enough to restore p53 activity) was achieved recently with a small-molecule filling the location of the mutated tyrosine side chain [36], four rescue mutants and three non-rescue mutants for the R273H cancer mutant [17], [18], two rescue mutants and one non-rescue mutant for the G245S cancer mutant (G245S_N239Y, G245S_T123P and G245S_E286D) [18], [37], two rescue mutants and one non-rescue mutant for the Y220C cancer mutant (Y220C_A138G, Y220C_L137R and Y220C_L114G), the superstable quadruple mutant M133L_V203A_N239Y_N268D [38], and stabilizing mutant N239Y [38].

### MD analysis

Conformational clustering was performed using the gromos algorithm [39] with GROMACS4.0.5 analysis software [40]. For each of the mutants, atomic coordinates were extracted at 10 ps intervals over the 30 ns MD simulation. The resulting 3000 structures were superimposed with respect to all C_α_ atoms to remove overall translation and rotation, then clustered at various RMSD cutoff values (i.e., 0.95, 1.05, and 1.15 Å) based on atomic coordinates of all C_α_ atoms of the protein. After calculating an RMSD-distance matrix of atomic positions between all pairs of MD snapshots in a trajectory, the gromos clustering algorithm counts the number of similar MD snapshots for which the calculated RMSD is less than or equal to the determined RMSD cutoff for each MD snapshot. The MD snapshot with the highest number of neighbors (e.g. the structure with the smallest RMSD between all the other structures in the cluster) is determined to be the center of the first cluster. Thus this structure is also referred to as the “cluster centroid.” Subsequently, this entire cluster (i.e. the cluster centroid and its neighbors) is eliminated from the pool of MD snapshots, and the same process is repeated until all MD snapshots are assigned to a cluster.

As another potential flexibility metric, root-mean-square-fluctuation of all C_α_ atoms of p53 in the trajectories were calculated using AMBER suite.

Two alternative clustering methods available in GROMACS package, namely single-linkage clustering and Jarvis-Patrick clustering, were also performed for comparison. A cutoff of 0.65 Å was used for single-linkage clustering. In Jarvis-Patrick clustering, the RMSD cutoff used to determine the number of nearest neighbors considered for Jarvis-Patrick algorithm was set to 0.80 Å, and the snaphots that have at least 3 identical nearest neighbors were assigned to the same cluster.

### Biological assays

**Figure 2:**
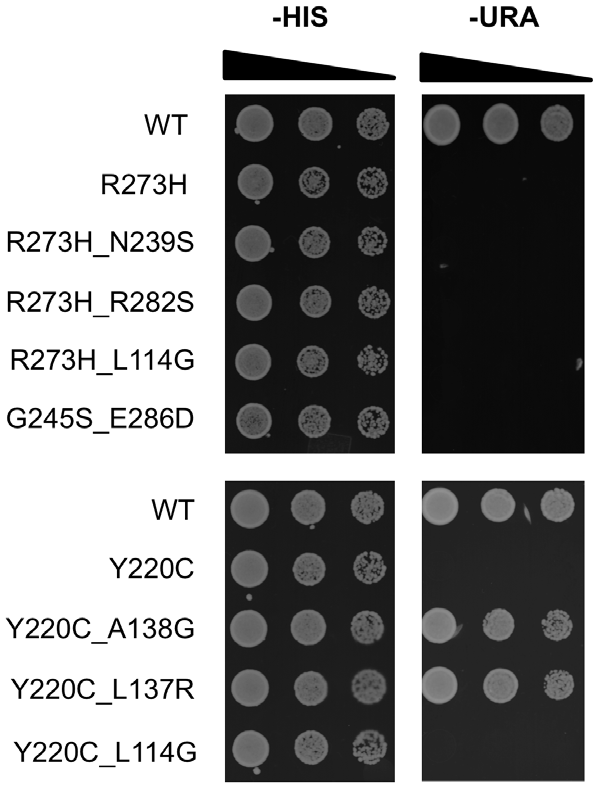
Biological validation of p53 cancer rescue mutations. A p53 tester yeast strain expressing the *URA3* gene under control of a p53-dependent promoter was transformed with centromeric plasmids (*HIS3* selection marker) expressing either wild-type human p53, or the mutants R273H, R273H_N239S, R273H_R282S, R272H_L114G, G245S_E286D, Y220C, Y220C_A138G, Y220C_L137R, and Y220C_L114G. Serial dilutions cells (10,000; 2,000; and 400) grown to mid-log phase were spotted onto agar plates lacking either histidine or uracil. Plates were incubated for 2 days at 37°C. Growth on plates lacking uracil is dependent on expression of the *URA3* gene and is a measure of p53 activity, while the growth on plates lacking histidine is selective for the presence of the plasmid.

Functional activities of rescue and non-rescue mutants for which no published experimental p53 activity result exists (R273H_N239S, R273H_R282S, R273H_L114G, G245S_E286D, Y220C_A138G, Y220C_L137R and Y220C_L114G) were verified using yeast assays (Figure 2). Wild-type p53 and relevant cancer mutants R273H and Y220C were also included in the assays as controls. For this purpose, p53-tester yeast strain RBy379 (1cUASp53::URA3 his3Δ200 a/alpha) [17], [41] expressing the *URA3* gene under control of a p53-dependent promoter was transformed with centromeric pTW300 plasmids [41] (*HIS3* selection marker) expressing either wild-type human p53 or the mutants indicated under control of the ADH1 promoter. Yeast strains were grown in YEPD (10% yeast extract, 20% pepton, 20% dextrose) and transformed with the relevant plasmids using a LiAc-based transformation protocol [42]. Transformants were selected on SC plates lacking histidine and incubated at 30°C. Serial dilutions of mid-log phase cells (10,000; 2,000; and 400 cells) were spotted onto agar plates lacking either histidine or uracil. Plates were incubated for 2 days at 37°C. The growth on plates lacking histidine is selective only for the presence of the plasmid, while growth on plates lacking uracil is dependent on expression of the *URA3* gene and is a measure of p53 activity [43].

## Results

In order to compare the dynamical effects of different mutations on p53, MD-generated trajectories of various p53 mutants were clustered based on overall structural similarity. Explicitly solvated MD simulations were run in the isothermal-isobaric (NPT) ensemble for 30 ns, after which RMSD-based clustering was performed on the resulting trajectories with the gromos clustering algorithm [39]. The RMSD distance matrix was computed in a pairwise fashion over all of the alpha carbons for each structure extracted every 10 ps from a particular trajectory (i.e., 3000 structures representing each trajectory). A large range of RMSD cutoff values were tested, including 0.95, 1.05, 1.15, 1.25, 1.35 and 1.60 Å. RMSD cutoffs larger than 1.15 Å caused loss of sensitivity of NOCs to the effect p53 mutations. The low optimal RMSD cutoff is an indication of a well-behaved system sampling configurations within a single energetically low-lying substate, as well as a reflection of the small size and low flexibility of the p53 core domain. The NOCs observed for p53 wild-type and its various mutants using a cutoff of 1.15 Å are shown in Tables 1 through [Table pcbi-1002238-t002] 3. Clustering results at several other RMSD cutoff values are presented in Table S1.

**Table 1:** Number of clusters (NOC) at 1.15 Å RMSD cutoff for the MD simulations and experimental thermodynamic stability data for wild-type p53 and its cancer mutants.

	Functional activity	NOC	Thermodynamic stability (kcal/mol)
wt	A	21	0^a^
**R273H**	I	27	0.45±0.03^a^
**R249S**	I	30	1.92±0.04^a^
**Y220C**	I	32	3.98±0.06^a^
**G245S**	I	32	1.21±0.03^a^
**R282W**	I	36	3.30±0.10^a^
**R248Q**	I	40	1.87±0.09^a^
**R175H**	I	42	3.52±0.06^a^

^*a*^Ref 23. Cancer mutants are typed in bold letters. A: active, I: inactive.

**Table 2 pcbi-1002238-t002:** Number of clusters (NOC) at 1.15 Å RMSD cutoff for the MD simulations of wild-type p53 and its functional and nonfunctional mutants.

	Relevant cancer mutant	Functional activity	NOC
wt		A	21
*R273H_N263V*	**R273H**	A	19
*R273H_N200Q_D208T*	**R273H**	A	19
*R273H_N235K_N239Y*	**R273H**	A	23
*R273H_S240R*	**R273H**	A	25
**R273H**	**R273H**	I	27
R273H_N239S	**R273H**	I	28
R273H_R282S	**R273H**	I	31
R273H_L114G	**R273H**	I	31
*G245S_N239Y*	**G245S**	A	15
**G245S**	**G245S**	I	32
*G245S_T123P*	**G245S**	A	36
G245S_E286D	**G245S**	I	45
*Y220C_A138G*	**Y220C**	A	21
*Y220C_L137R*	**Y220C**	A	29
**Y220C**	**Y220C**	I	32
Y220C_L114G	**Y220C**	I	55

Cancer mutants are typed in bold letters, rescue mutants are italicized, and non-rescue mutants are underlined. Wild-type p53 is presented as the first line of table. Cancer mutants and their relevant second-site mutants are grouped according to their relevant cancer mutants and sorted in ascending number of clusters in each group. A: active, I: inactive. The thermodynamic stability of G245S_N239Y mutant is given in Ref 36 to be −0.14 kcal/mol.

**Table 3:** Number of clusters (NOC) at 1.15 Å RMSD cutoff for the MD simulations and experimental thermodynamic stability data for wild-type p53 and its more-stabilized forms.

	NOC	Thermodynamic stability (kcal/mol)
wt	21	0^a^
N239Y	19	−1.49^b^
M133L_V203A_N239Y_N268D (first 30 ns of MD simulation)	25	−2.65^b^
M133L_V203A_N239Y_N268D (second 30 ns of MD simulation)	9	−2.65^b^

^*a*^Ref 23.

^*b*^Ref 37. Both mutants are functionally active.

### Cancer mutants are more flexible than wild-type p53

The number of clusters was significantly higher for the cancer mutants (in bold in Table 1) compared to the wild-type p53, which suggests that oncogenic mutations increase the overall plasticity of the p53 core domain. This result is consistent with Rauf *et al.* [44], who investigated the effects of different oncogenic mutations on the flexibility of the p53 DNA-binding domain using a graph theoretical approach. Here, the oncogenic property for all four structural classes of p53 mutants has been quantified by a single metric.

The flexibility of the structural and zinc-binding domain mutant R175H, as characterized by the number of clusters, was remarkably higher compared to the wild-type and other cancer mutants (Table 1). The especially high degree of flexibility exhibited by this system may explain why so far it has not been possible to rescue the R175H mutant with second-site suppressor mutations, even though all possible single point core domain mutations of R175H were tested exhaustively for p53 function [18].

The number-of-clusters (NOC) metric presented here correctly locates the DNA-contact mutant R273H as the closest cancer mutant to the wild-type in terms of structural variability over the 30 ns trajectories. R273H has been previously demonstrated to be the easiest to rescue among the most-frequent cancer mutants [23]. The thermodynamic stability of the R273H mutant is the closest to the wild-type p53 among the 19 cancer mutants considered by Bullock *et al* [23]. The number of rescue mutants known to reactivate R273H mutant is large compared to few or no rescue mutants known to reactivate each of the other hot spot cancer mutants [17], [18], [37], [38].

### Rescue mutations decrease the flexibility of cancer mutants

Comparison of the number of clusters for the R273H rescue mutants (in italics in Table 2) with those for the R273H cancer mutant (in bold in Table 2) indicated a significant decrease in flexibility for the rescue mutants. The restoration of stability to the protein was especially remarkable in the case of the R273H_N263V and R273H_N200Q_D208T rescue mutants, for which the number of clusters was even lower than the wild-type p53. Although thermodynamic stability data is not available for these rescue mutations, our results suggest that such values would be lower than wild-type p53.

As there are no single point mutations that can strongly rescue cancer mutants R175H, R248Q, R249S or R282W, the generality of this finding was tested on rescue mutants for which experimental data is available (in italics in Table 2). More specifically, the known rescue mutants G245S_N239Y and G245S_T123P were considered for the class of structural mutants in the DNA binding surface. Similarly, two rescue mutants for the β-sandwich mutant Y220C, Y220C_A138G and Y220C_L137R, were investigated with the same approach. Functional activity of the latter two rescue mutants, for which no published experimental p53 activity results exist, was verified using yeast assays and depicted in Figure 2. Three out of these four rescue mutants showed decreased flexibility compared to their relevant cancer mutant (Table 2). The only exception in this test set was G245S_T123P, which exhibited more clusters than its cancer mutant G245S.

### Nonrescue mutations introduce even more flexibility to cancer mutants

As negative controls, we tested experimentally confirmed non-rescue second-site mutations (underlined in Table 2) of relevant cancer mutants with the same approach. Functional inactivity of all the nonrescue mutants was verified using yeast assays and depicted in Figure 2. All non-rescue mutants that we simulated were more flexible, as compared to their relevant cancer mutant, indicating destabilization introduced to the cancer mutant by these second-site mutations. Thus, our method can successfully discriminate rescue mutants from non-rescue mutants.

### More stable p53 mutants follow a similar trend

We extended the same analysis on stabilizing mutant N239Y and the “superstable” quadruple mutant M133L_V203A_N239Y_N268D [38] (Table 3). The N239Y mutant exhibited a significant decrease in flexibility compared to the wild-type. In contrast, the superstable mutant did not follow the same trend (Table 3). This may be due to the need for longer relaxation in MD simulations in order to account for the greater extent of structural change introduced by four point mutations. To explore this hypothesis, we extended the quadruple mutant MD simulation for an additional 30 ns (for a total of 60 ns of production dynamics). In the second 30 ns, its number of clusters decreased significantly to a value much lower than that of the wild-type p53 as hypothesized.

### At least 30 ns MD simulation is required

**Figure 3:**
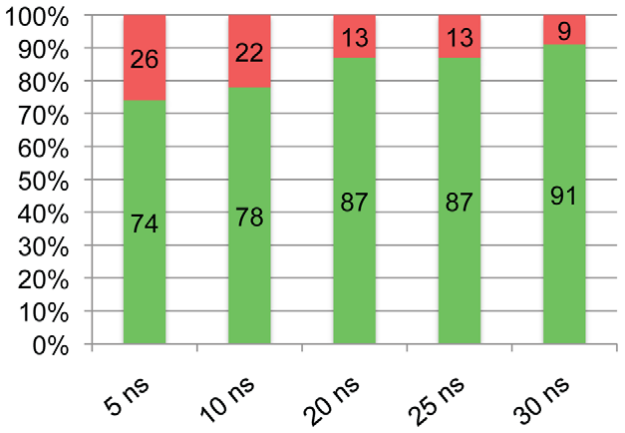
Time evolution of the predictive ability of flexibility metric during molecular dynamics (MD) simulations. Shorter segments of MD trajectories were analyzed in order to find out the predictive ability of the flexibility metric in terms of discriminating between the functional and nonfunctional forms of p53 mutants. The percentage of succesful predictions using the number of clusters metric for the p53 mutants calculated at each of 5, 10, 20, 25 and 30 ns of MD simulations are plotted in the form of a column graph. The green and red column bars at each MD segment represents the percentage of successful and unsuccessful predictions, respectively. The exact percentage values are printed in each column bar.

The data presented in tables 1–3 relies on the full 30 ns trajectories of p53. In order to determine what is the shortest MD simulation necessary to discriminate between the functional and nonfunctional forms of p53 mutants, shorter segments of the full production MD trajectories were analyzed. At the RMSD cutoff of 1.15 Å, the number of clusters for each p53 mutant calculated at 5, 10, 20, 25 and 30 ns of MD simulations were separately depicted as column graphs in Figure S2. In this set of graphs, active and inactive p53 mutants were grouped and designated with a green arrow and a red arrow, respectively. In Figure 3, the percentage of mutants for which p53 function was correctly predicted by our flexibility metric are depicted for 5, 10, 20, 25 and 30 ns of MD simulations. The success of function prediction increased from 74% to 91% while our simulation time increased from 5 ns to 30 ns. This analysis indicated that at least 30 ns of MD simulation is required for a successful prediction of function of p53 mutants.

### NOC metric correlates with experimental thermodynamic stability data

The thermodynamic stability values of several p53 cancer mutants are available in the literature (Table 1), as measured by urea-induced unfolding experiments at 283 K [23], [37], [38]. There are no comparable experimental data for the rescue or non-rescue mutants, which were not included in this part of the analysis. All cancer mutants evaluated experimentally exhibit differential experimental destabilization compared to wild-type p53 (Table 1).

**Figure 4:**
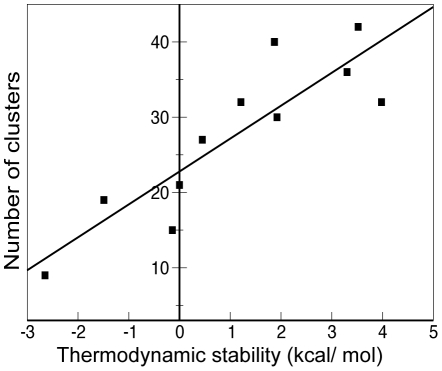
The correlation of the number of clusters and the thermodynamic stability for p53 mutants. The number of clusters is computed at RMSD cutoff of 1.15 Å. The predicted linear regression line is also shown (y = 4.37×+22.79). r^2^ value is 0.77.

Figure 4 depicts the correlation between the available thermodynamic stability values of p53 cancer mutants and the number of clusters observed in the MD simulations at the RMSD cutoff of 1.15 Å (r^2^ = 0.77). The r^2^ values for p53 single mutants at RMSD cutoff values of 0.95 Å and 1.05 Å are both 0.74. The number of clusters at these cutoffs for each mutant is tabulated in Table S1. The number of clusters in the second 30 ns MD simulation of the superstable mutant was used for this correlation analysis. If the NOCs in the initial 30 ns MD simulation of the superstable mutants was used instead, the r^2^ value decreased from 0.77 to 0.55. If the average of the two was used, the r^2^ value became 0.70. Excluding the superstable mutant form the data set gave an r^2^ value of 0.66. Remarkably, the number of clusters metric alone explains about three-quarters of the variance in experimentally measured thermodynamic stability values of p53 mutants.

To compare the NOC metric with another simple flexibility metric, the root-mean-square-fluctuation (RMSF) values of all C_α_ atoms of p53 were calculated using the AMBER suite for each mutant (Table S2). The correlation of the RMSF values with the thermodynamic data gave an r^2^ value of 0.62, which showed the superiority of the NOC method comparing to its r^2^ value of 0.77. To compare the performance of other clustering methods, single-linkage clustering and Jarvis-Patrick clustering were performed on the MD trajectories (Table S3). Both methods resulted in a lower correlation with the thermodynamic stability values versus the RMSD-based clustering method, with r^2^ values of 0.44 and 0.45, respectively

## Discussion

p53 is an inherently unstable protein, as reflected by its low melting temperature of ∼42–44°C [45]. It has been shown that the main reason of p53 instability is neither poor packing density nor the presence of unusually large void volumes in the protein. Instead, an analysis of the solution structure of p53 core domain obtained by NMR has revealed several reasons for instability of p53 [46]. First, this study indicated that p53 has buried hydroxyl and sulfhydryl groups that form sub-optimal hydrogen-bonding networks. Second, high flexibility of loop regions, especially of L1 loop, is observed in p53. Lastly, some buried tyrosine residues were found to be involved in temperature-dependent dynamic processes possibly indicating presence of alternative hydrogen-bonding networks in p53. Based on all of these factors, Canadillas *et al* concluded that “the p53 structure is more flexible than is apparent from crystal structures” [46].

In an effort to capture this intrinsic structural flexibility, we have focused on finding a computational method to measure the overall flexibility of the p53 core domain and the effect of mutations, be they cancer mutations, rescue mutations or non-rescue mutations, on the flexibility. This work presents a new method in which the number of structural clusters representing an explicitly solvated all-atom MD trajectory can be used as a single robust measure of overall flexibility in the p53 core domain. All hot-spot cancer mutants we studied demonstrated higher flexibility compared to the wild-type p53, in line with the results of an earlier graph-theoretical approach that assessed the flexibilities of wild-type p53 and several cancer mutants [44]. Testing rescue and non-rescue mutants for particular cancer mutants, the number of clusters for functional p53 mutants was found to differ significantly from the nonfunctional p53 mutants. Remarkably, the NOC metric is able to predict which second-site mutations may restore p53 activity to cancer mutants and which will leave p53 functionally defective.

It is also notable that such a simple metric reflecting system flexibility or entropy can account for three-quarters of the variance in experimentally measured thermodynamic stability values of p53 mutants. MD simulations thus promise to be a robust tool to predict thermodynamic stability of p53 mutants of interest. The NOC metric could further be used to discover new rescue mutants that restore p53 activity, and thus kill the cancer cell. Additionally, whether binding of a small-molecule can achieve enough stabilization to restore p53 function to cancer mutants could be tested with this metric. The computational cost of performing classical MD simulations could be decreased by using alternative methods such as accelerated MD, which may achieve increased sampling of conformational states over significantly shorter simulation timescales. Experimental efficiencies could be achieved through an integrated strategy that is guided by use of the NOC metric as a predictive measure for p53 function.

## Supporting Information

Figure S1 **Root mean square deviations (RMSD) of Cα atoms of p53 wild-type and mutant systems during simulations.**
(PDF)

Figure S2 **Time evolution of the number of clusters during molecular dynamics simulations.** Shorter segments of MD trajectories were analyzed in order to find out what is the shortest MD simulation necessary to discriminate between the functional and nonfunctional forms of p53 mutants. Number of clusters for the p53 mutants calculated at each of 5, 10, 20, 25 and 30 ns of MD simulations are graphed separately in the form of column graphs. Functionally active p53 mutants are grouped and designated with a green arrow while nonfunctional p53 mutants were designated with a red arrow. The clustering results at RMSD cutoff of 1.15 Å indicates that at least 30 ns of MD simulation is required.
(PDF)

Table S1 **The number of clusters at different RMSD cutoffs for p53 mutants.**
(DOC)

Table S2 **The root-mean-square-fluctuations for p53 mutants in MD trajectories.**
(DOC)

Table S3 **The number of clusters for p53 mutants in MD trajectories using single-linkage algorithm and Jarvis-Patrick algorithm.**
(DOC)

## References

[1] Levine AJ, Hu W, Feng Z (2006). The P53 pathway: what questions remain to be explored?. Cell Death Differ.

[2] Vogelstein B, Lane D, Levine AJ (2000). Surfing the p53 network.. Nature.

[3] Vousden KH, Lu X (2002). Live or let die: the cell's response to p53.. Nat Rev Cancer.

[4] Cho Y, Gorina S, Jeffrey PD, Pavletich NP (1994). Crystal structure of a p53 tumor suppressor-DNA complex: understanding tumorigenic mutations.. Science.

[5] Joerger AC, Fersht AR (2010). The tumor suppressor p53: from structures to drug discovery.. Cold Spring Harb Perspect Biol.

[6] Martins CP, Brown-Swigart L, Evan GI (2006). Modeling the therapeutic efficacy of p53 restoration in tumors.. Cell.

[7] Ventura A, Kirsch DG, McLaughlin ME, Tuveson DA, Grimm J (2007). Restoration of p53 function leads to tumour regression in vivo.. Nature.

[8] Feldser DM, Kostova KK, Winslow MM, Taylor SE, Cashman C (2010). Stage-specific sensitivity to p53 restoration during lung cancer progression.. Nature.

[9] Junttila MR, Karnezis AN, Garcia D, Madriles F, Kortlever RM (2010). Selective activation of p53-mediated tumour suppression in high-grade tumours.. Nature.

[10] Bykov VJ, Issaeva N, Selivanova G, Wiman KG (2002). Mutant p53-dependent growth suppression distinguishes PRIMA-1 from known anticancer drugs: a statistical analysis of information in the National Cancer Institute database.. Carcinogenesis.

[11] Bykov VJ, Issaeva N, Shilov A, Hultcrantz M, Pugacheva E (2002). Restoration of the tumor suppressor function to mutant p53 by a low-molecular-weight compound.. Nat Med.

[12] Bykov VJ, Issaeva N, Zache N, Shilov A, Hultcrantz M (2005). Reactivation of mutant p53 and induction of apoptosis in human tumor cells by maleimide analogs.. J Biol Chem.

[13] Foster BA, Coffey HA, Morin MJ, Rastinejad F (1999). Pharmacological rescue of mutant p53 conformation and function.. Science.

[14] North S, Pluquet O, Maurici D, El-Ghissassi F, Hainaut P (2002). Restoration of wild-type conformation and activity of a temperature-sensitive mutant of p53 (p53(V272M)) by the cytoprotective aminothiol WR1065 in the esophageal cancer cell line TE-1.. Mol Carcinog.

[15] Wieczorek AM, Waterman JL, Waterman MJ, Halazonetis TD (1996). Structure-based rescue of common tumor-derived p53 mutants.. Nat Med.

[16] Brachmann RK, Yu K, Eby Y, Pavletich NP, Boeke JD (1998). Genetic selection of intragenic suppressor mutations that reverse the effect of common p53 cancer mutations.. EMBO J.

[17] Baroni TE, Wang T, Qian H, Dearth LR, Truong LN (2004). A global suppressor motif for p53 cancer mutants.. Proc Natl Acad Sci U S A.

[18] Baronio R, Danziger SA, Hall LV, Salmon K, Hatfield GW (2010). All-codon scanning identifies p53 cancer rescue mutations.. Nucleic Acids Res.

[19] Joerger AC, Allen MD, Fersht AR (2004). Crystal structure of a superstable mutant of human p53 core domain. Insights into the mechanism of rescuing oncogenic mutations.. J Biol Chem.

[20] Joerger AC, Ang HC, Fersht AR (2006). Structural basis for understanding oncogenic p53 mutations and designing rescue drugs.. Proc Natl Acad Sci U S A.

[21] Joerger AC, Ang HC, Veprintsev DB, Blair CM, Fersht AR (2005). Structures of p53 cancer mutants and mechanism of rescue by second-site suppressor mutations.. J Biol Chem.

[22] Ang HC, Joerger AC, Mayer S, Fersht AR (2006). Effects of common cancer mutations on stability and DNA binding of full-length p53 compared with isolated core domains.. J Biol Chem.

[23] Bullock AN, Henckel J, Fersht AR (2000). Quantitative analysis of residual folding and DNA binding in mutant p53 core domain: definition of mutant states for rescue in cancer therapy.. Oncogene.

[24] Case DA, Darden T, Cheatham TE, Simmerling CL, Wang J (2006). Amber9.

[25] Chen VB, Arendall WB, Headd JJ, Keedy DA, Immormino RM (2010). MolProbity: all-atom structure validation for macromolecular crystallography.. Acta Crystallogr D Biol Crystallogr.

[26] Rodriguez R, Chinea G, Lopez N, Pons T, Vriend G (1998). Homology modeling, model and software evaluation: three related resources.. Bioinformatics.

[27] Pang YP (1999). Novel zinc protein molecular dynamics simulations: Steps toward antiangiogenesis for cancer treatment.. J Mol Model.

[28] Jorgensen WL, Chandrasekhar J, Madura JD, Impey RW, Klein ML (1983). Comparison of Simple Potential Functions for Simulating Liquid Water.. J Chem Phys.

[29] Hornak V, Abel R, Okur A, Strockbine B, Roitberg A (2006). Comparison of multiple amber force fields and development of improved protein backbone parameters.. Proteins Struct Funct Bioinf.

[30] Martyna GJ, Tobias DJ, Klein ML (1994). Constant-Pressure Molecular-Dynamics Algorithms.. J Chem Phys.

[31] Feller SE, Zhang YH, Pastor RW, Brooks BR (1995). Constant-Pressure Molecular-Dynamics Simulation - the Langevin Piston Method.. J Chem Phys.

[32] Darden T, Perera L, Li L, Pedersen L (1999). New tricks for modelers from the crystallography toolkit: the particle mesh Ewald algorithm and its use in nucleic acid simulations.. Structure.

[33] Ryckaert J-P, Ciccotti G, Berendsen HJC (1977). Numerical integration of the cartesian equations of motion of a system with constraints: Molecular dynamics of n-alkanes.. J Comput Phys.

[34] Miyamoto S, Kollman PA (1992). Settle: An analytical version of the SHAKE and RATTLE algorithm for rigid water models.. J Comput Chem.

[35] Phillips JC, Braun R, Wang W, Gumbart J, Tajkhorshid E (2005). Scalable molecular dynamics with NAMD.. J Comput Chem.

[36] Boeckler FM, Joerger AC, Jaggi G, Rutherford TJ, Veprintsev DB (2008). Targeted rescue of a destabilized mutant of p53 by an in silico screened drug.. Proc Natl Acad Sci U S A.

[37] Nikolova PV, Wong KB, DeDecker B, Henckel J, Fersht AR (2000). Mechanism of rescue of common p53 cancer mutations by second-site suppressor mutations.. EMBO J.

[38] Nikolova PV, Henckel J, Lane DP, Fersht AR (1998). Semirational design of active tumor suppressor p53 DNA binding domain with enhanced stability.. Proc Natl Acad Sci U S A.

[39] Daura X, Gademann K, Jaun B, Seebach D, van Gunsteren WF (1999). Peptide folding: When simulation meets experiment.. Angew Chem-Int Edit.

[40] Hess B, Kutzner C, van der Spoel D, Lindahl E (2008). GROMACS 4: Algorithms for highly efficient, load-balanced, and scalable molecular simulation.. J Chem Theory Comput.

[41] Sikorski RS, Hieter P (1989). A system of shuttle vectors and yeast host strains designed for efficient manipulation of DNA in Saccharomyces cerevisiae.. Genetics.

[42] Hill J, Donald KA, Griffiths DE (1991). DMSO-enhanced whole cell yeast transformation.. Nucleic Acids Res.

[43] Danziger SA, Baronio R, Ho L, Hall L, Salmon K (2009). Predicting Positive p53 Cancer Rescue Regions Using Most Informative Positive (MIP) Active Learning.. PLOS Comput Biol.

[44] Rauf SMA, Ismael M, Sahu KK, Suzuki A, Sahnoun R (2009). A graph theoretical approach to the effect of mutation on the flexibility of the DNA binding domain of p53 protein.. Chem Papers.

[45] Bullock AN, Henckel J, DeDecker BS, Johnson CM, Nikolova PV (1997). Thermodynamic stability of wild-type and mutant p53 core domain.. Proc Natl Acad Sci U S A.

[46] Canadillas JM, Tidow H, Freund SM, Rutherford TJ, Ang HC (2006). Solution structure of p53 core domain: structural basis for its instability.. Proc Natl Acad Sci U S A.

