# Supplementary material for: Ensemble-Based Computational Approach Discriminates Functional Activity of p53 Cancer and Rescue Mutants
Source: PLoS Comput Biol. 2011 Oct 20;7(10):e1002238. doi: 10.1371/journal.pcbi.1002238 (PMC3197647; doi:10.1371/journal.pcbi.1002238)
Supplement: Figure S1 — Root mean square deviations (RMSD) of Cα atoms of p53 wild-type and mutant systems during simulations. (PDF) [file pcbi.1002238.s001.pdf]

Figure S1. Root mean square deviations (RMSD) of C_α atoms of p53 wild-type and mutant systems during simulations

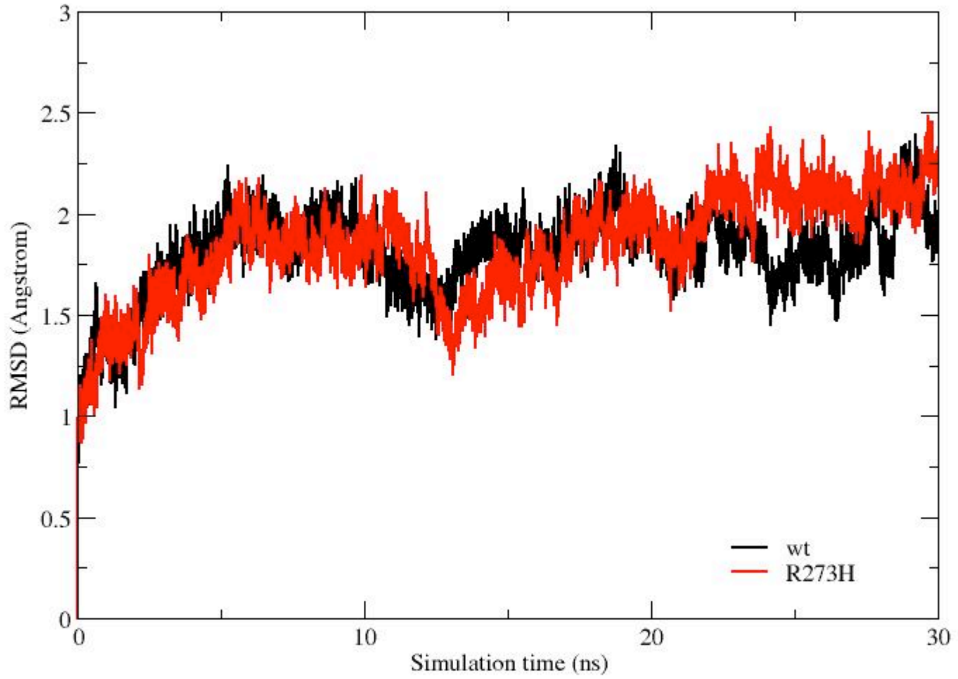

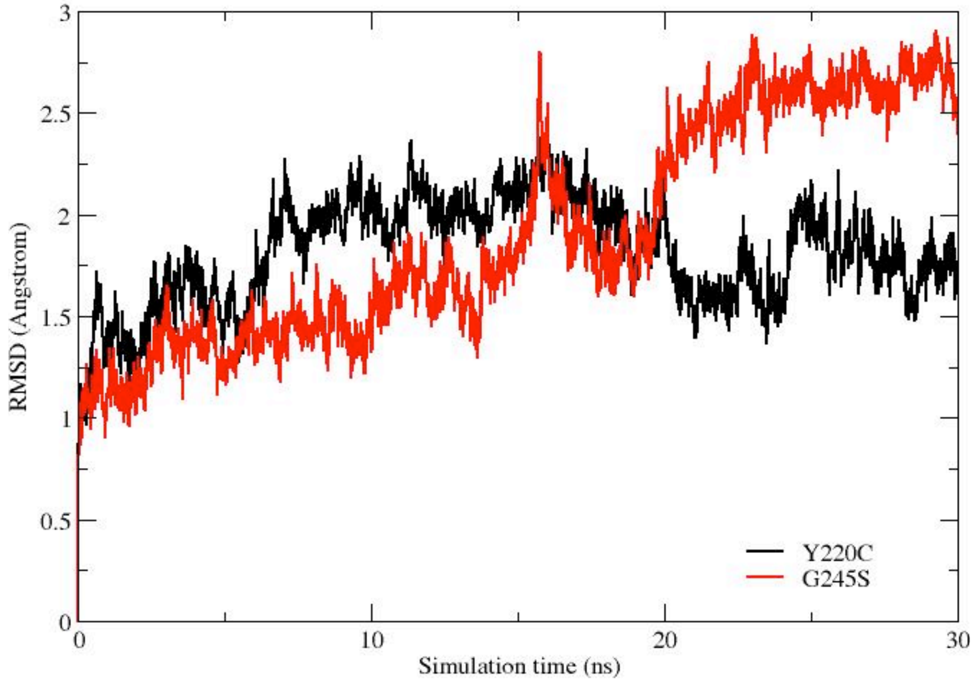

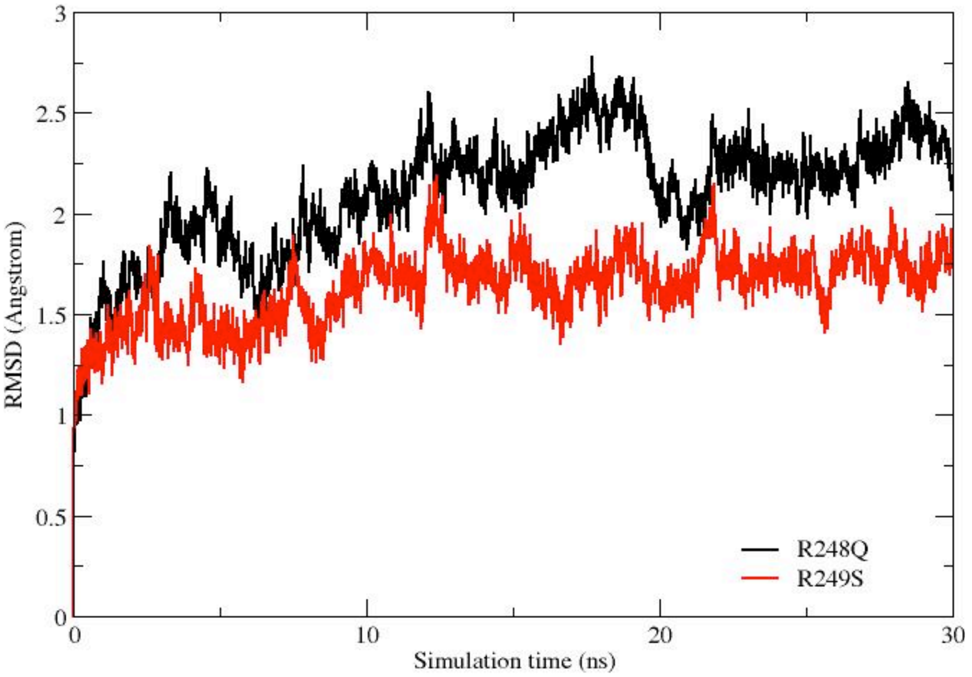
RMSD (Angstrom)
R248Q
R249S
Simulation time (ns)

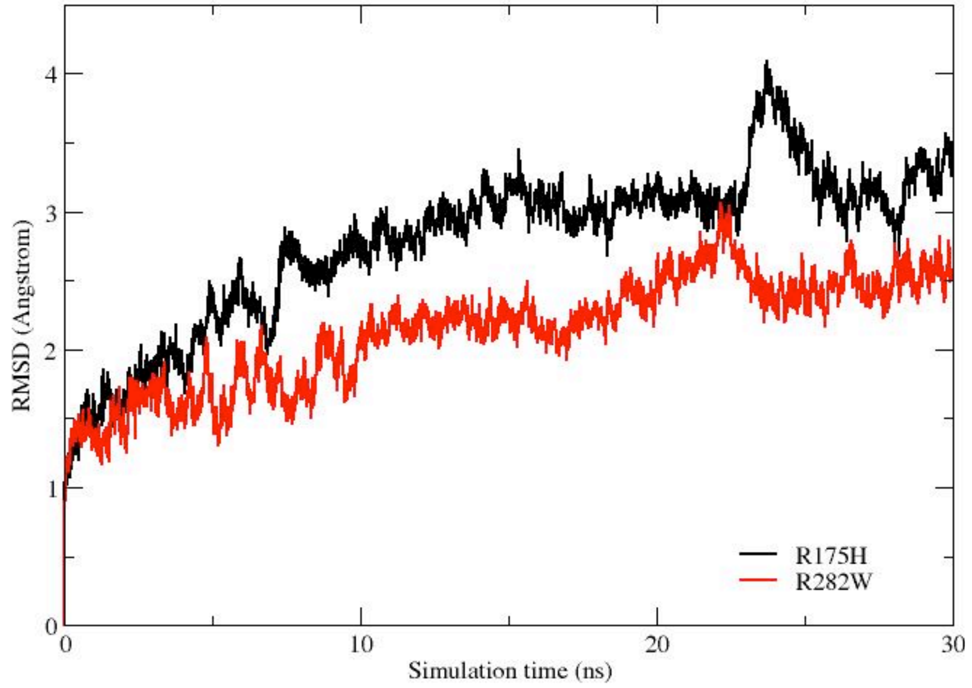
RMSD (Angstrom)
R175H
R282W
Simulation time (ns)

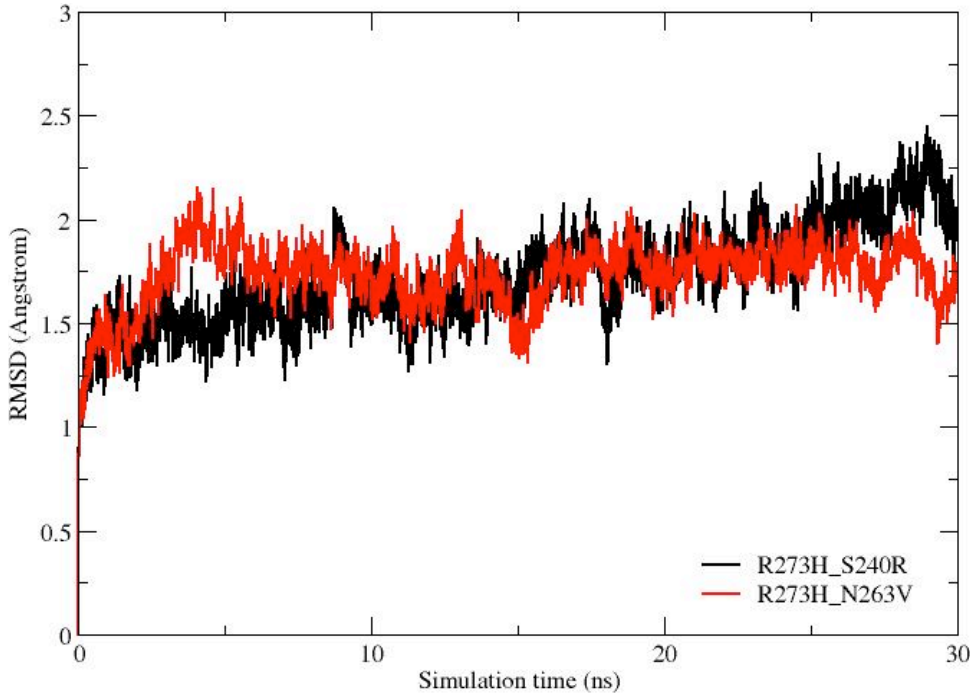
RMSD (Angstrom)
Simulation time (ns)
R273H_S240R
R273H_N263V
0
0.5
1
1.5
2
2.5
3
0
10
20
30

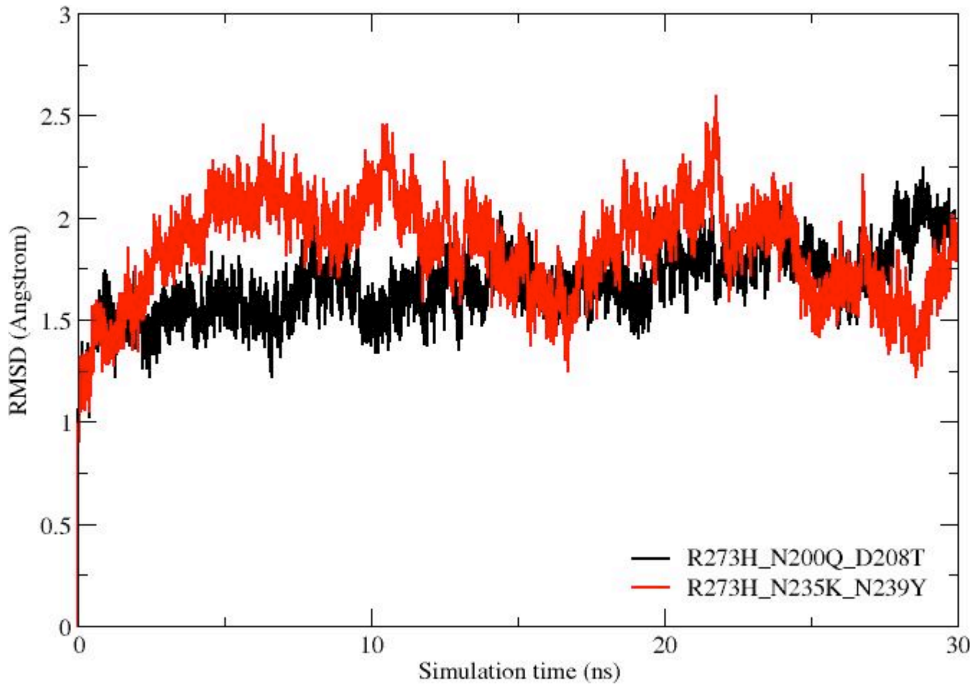
RMSD (Angstrom)
Simulation time (ns)
R273H_N200Q_D208T
R273H_N235K_N239Y
0
0.5
1
1.5
2
2.5
3
0
10
20
30

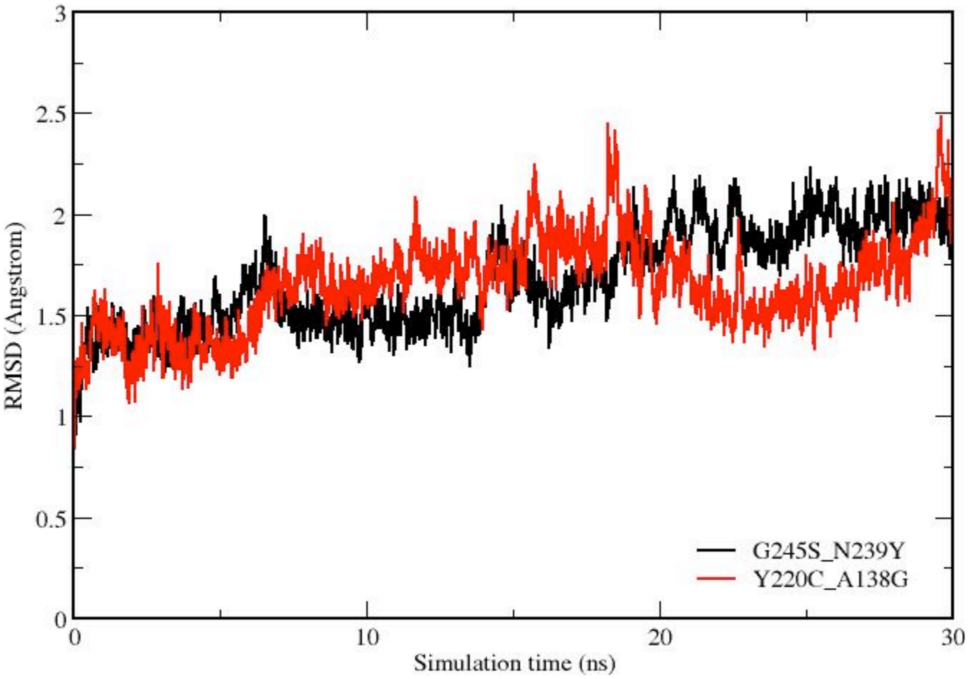
RMSD (Angstrom)
Simulation time (ns)
G245S_N239Y
Y220C_A138G
0
0.5
1
1.5
2
2.5
3
0
10
20
30

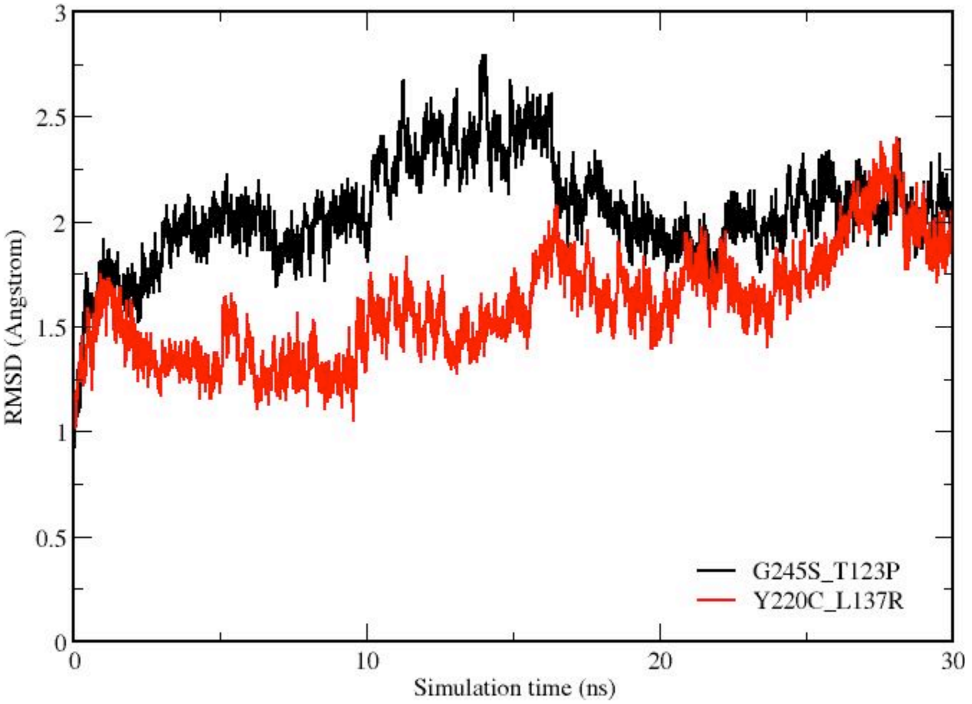
RMSD (Angstrom)
Simulation time (ns)
G245S_T123P
Y220C_L137R
0
0.5
1
1.5
2
2.5
3
0
10
20
30

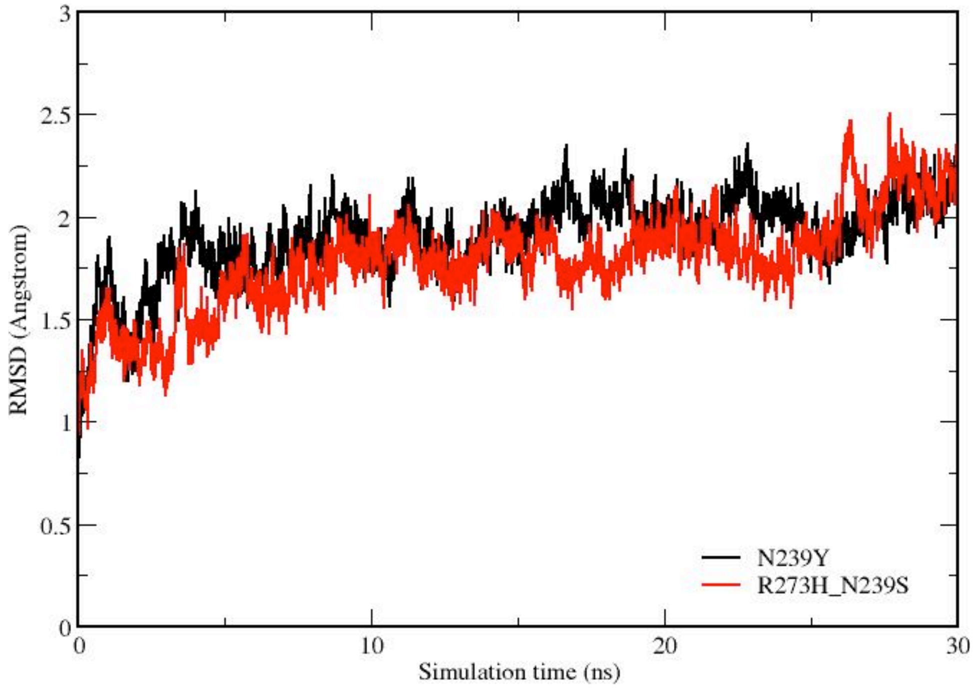
RMSD (Angstrom)
Simulation time (ns)
N239Y
R273H_N239S
0
0.5
1
1.5
2
2.5
3
10
20
30

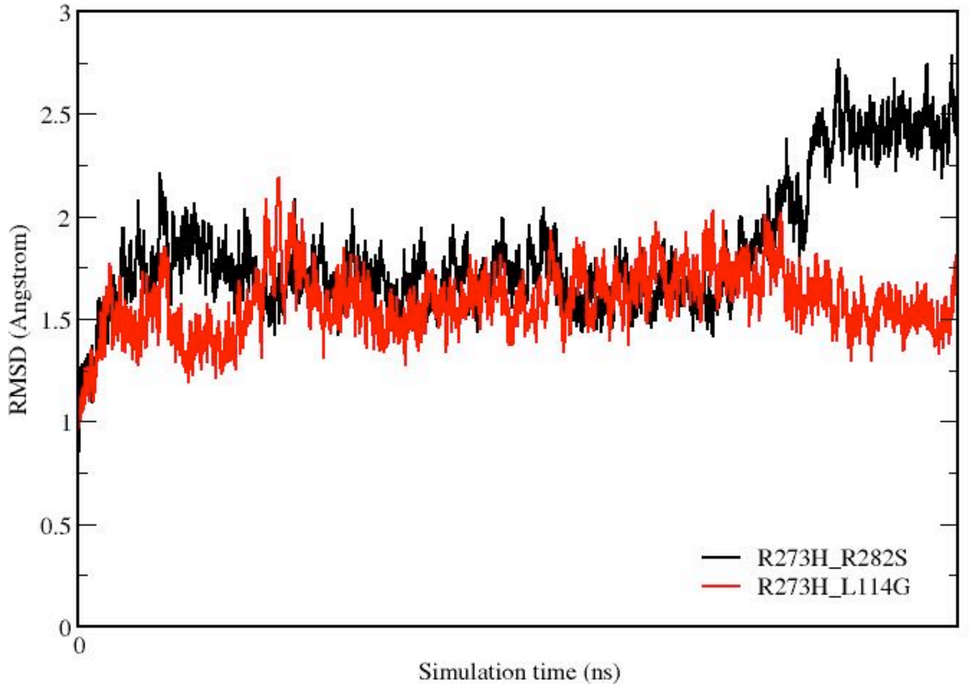
RMSD (Angstrom)
Simulation time (ns)
R273H_R282S
R273H_L114G
0
0.5
1
1.5
2
2.5
3

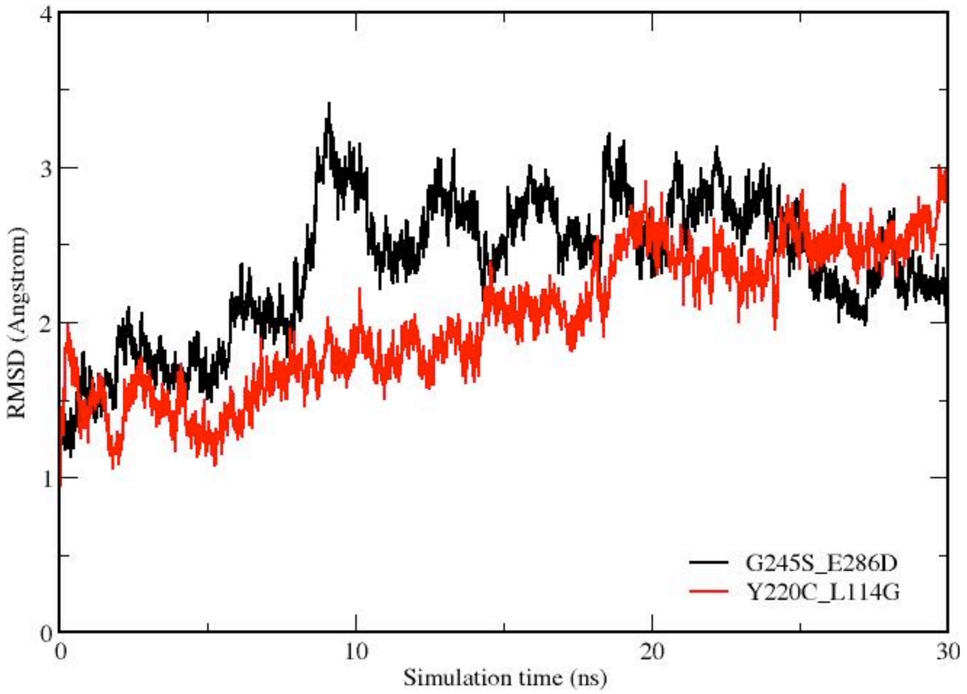
RMSD (Angstrom)
Simulation time (ns)
G245S_E286D
Y220C_L114G
0
1
2
3
4
0
10
20
30

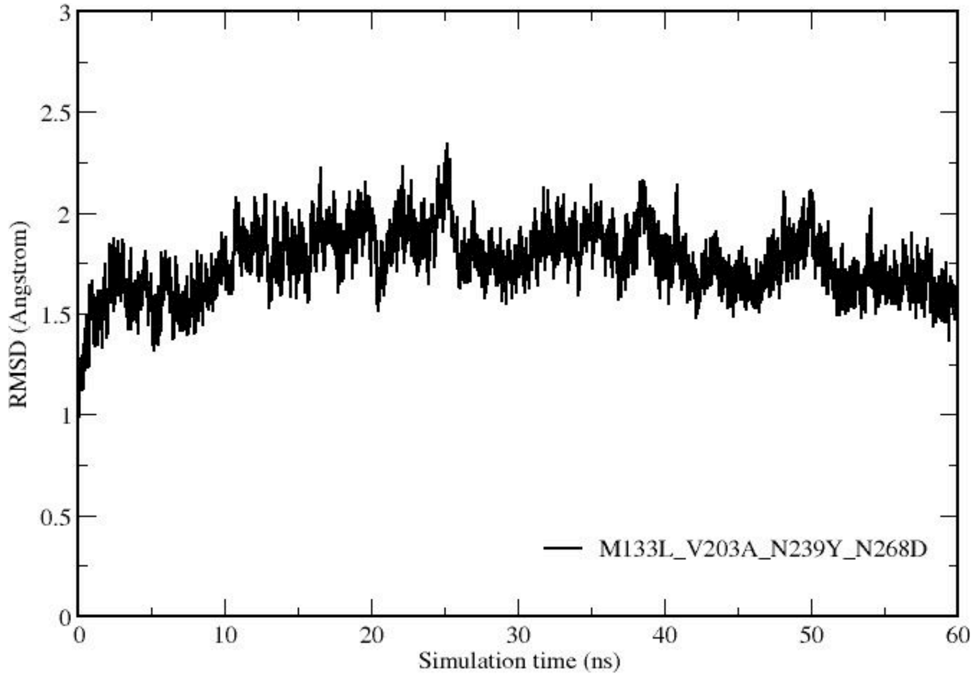
RMSD (Angstrom)
Simulation time (ns)
M133L_V203A_N239Y_N268D
0
0.5
1
1.5
2
2.5
3
0
10
20
30
40
50
60
