# Supplementary material for: Ensemble-Based Computational Approach Discriminates Functional Activity of p53 Cancer and Rescue Mutants
Source: PLoS Comput Biol. 2011 Oct 20;7(10):e1002238. doi: 10.1371/journal.pcbi.1002238 (PMC3197647; doi:10.1371/journal.pcbi.1002238)
Supplement: Figure S2 — Time evolution of the number of clusters during molecular dynamics simulations. Shorter segments of MD trajectories were analyzed in order to find out what is the shortest MD simulation necessary to discriminate between the functional and nonfunctional forms of p53 mutants. Number of clusters for the p53 mutants calculated at each of 5, 10, 20, 25 and 30 ns of MD simulations are graphed separately in the form of column graphs. Functionally active p53 mutants are grouped and designated with a green arrow while nonfunctional p53 mutants were designated with a red arrow. The clustering results at RMSD cutoff of 1.15 Å indicates that at least 30 ns of MD simulation is required. (PDF) [file pcbi.1002238.s002.pdf]

Figure S2. Time evolution of the number of clusters during molecular dynamics simulations.

Shorter segments of MD trajectories were analyzed in order to find out what is the shortest MD simulation necessary to discriminate between the functional and nonfunctional forms of p53 mutants. Number of clusters for the p53 mutants calculated at each of 5, 10, 20, 25 and 30 ns of MD simulations are graphed separately in the form of column graphs. Functionally active p53 mutants are grouped and designated with a green arrow while nonfunctional p53 mutants were designated with a red arrow.The clustering results at RMSD cutoff of 1.15 Å indicates that at least 30 ns of MD simulation is required.

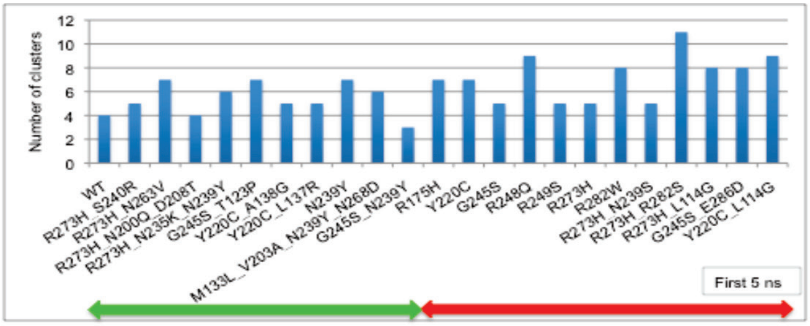
Number of clusters
0
2
4
6
8
10
12
WT
R273H_S240R
R273H_N263V
R273H_N200Q_D208T
R273H_N235K_N239Y
G245S_T123P
Y220C_A138G
Y220C_L137R
N239Y
M133L_V203A_N239Y_N268D
G245S_N239Y
R175H
Y220C
G245S
R248Q
R249S
R273H
R282W
R273H_N239S
R273H_R282S
R273H_L114G
G245S_E286D
Y220C_L114G
First 5 ns

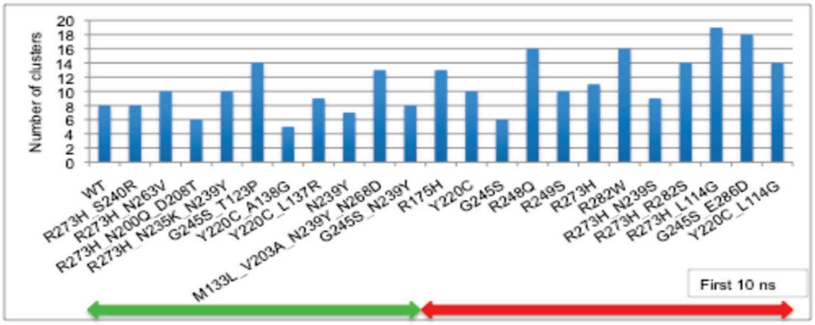
Number of clusters
0
2
4
6
8
10
12
14
16
18
20
WT
R273H_S240R
R273H_N263V
R273H_N200Q_D208T
R273H_N235K_N239Y
G245S_T123P
Y220C_A138G
Y220C_L137R
N239Y
M133L_V203A_N239Y_N268D
G245S_N239Y
R175H
Y220C
G245S
R248Q
R249S
R273H
R282W
R273H_N239S
R273H_R282S
R273H_L114G
G245S_E286D
Y220C_L114G
First 10 ns

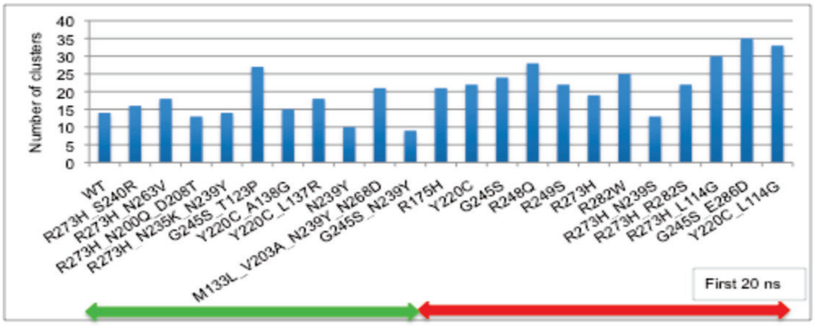
Number of clusters
0
5
10
15
20
25
30
35
40
WT
R273H_S240R
R273H_N263V
R273H_N200Q_D208T
R273H_N235K_N239Y
G245S_T123P
Y220C_A138G
Y220C_L137R
N239Y
M133L_V203A_N239Y_N268D
G245S_N239Y
R175H
Y220C
G245S
R248Q
R249S
R273H
R282W
R273H_N239S
R273H_R282S
R273H_L114G
G245S_E286D
Y220C_L114G
First 20 ns

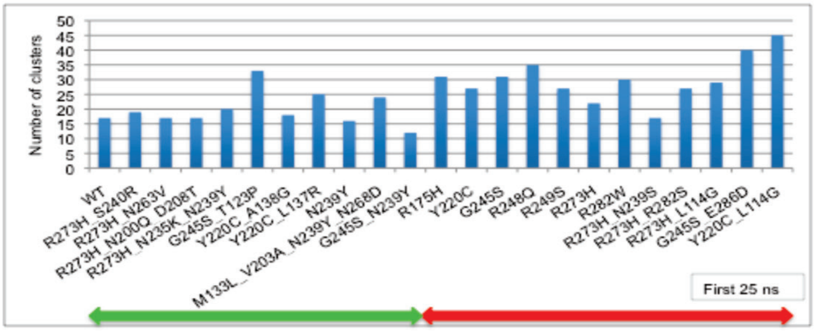
Number of clusters
0
5
10
15
20
25
30
35
40
45
50
WT
R273H_S240R
R273H_N263V
R273H_N200Q_D208T
R273H_N235K_N239Y
G245S_T123P
Y220C_A138G
Y220C_L137R
N239Y
M133L_V203A_N239Y_N268D
G245S_N239Y
R175H
Y220C
G245S
R248Q
R249S
R273H
R282W
R273H_N239S
R273H_R282S
R273H_L114G
G245S_E286D
Y220C_L114G
First 25 ns

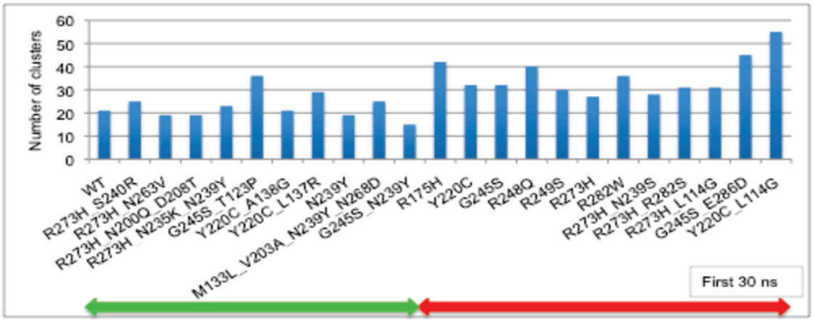
Number of clusters
0
10
20
30
40
50
60
WT
R273H_S240R
R273H_N263V
R273H_N200Q_D208T
R273H_N235K_N239Y
G245S_T123P
Y220C_A138G
Y220C_L137R
N239Y
M133L_V203A_N239Y_N268D
G245S_N239Y
R175H
Y220C
G245S
R248Q
R249S
R273H
R282W
R273H_N239S
R273H_R282S
R273H_L114G
G245S_E286D
Y220C_L114G
First 30 ns
