# Supplementary material for: Ensemble-Based Computational Approach Discriminates Functional Activity of p53 Cancer and Rescue Mutants
Source: PLoS Comput Biol. 2011 Oct 20;7(10):e1002238. doi: 10.1371/journal.pcbi.1002238 (PMC3197647; doi:10.1371/journal.pcbi.1002238)
Supplement: Table S1 — The number of clusters at different RMSD cutoffs for p53 mutants. (DOC) [file pcbi.1002238.s003.doc]

Table S1. The number of clusters at different RMSD cutoffs for p53 mutants

|  | Number of clusters at 0.95 Å cutoff | Number of clusters at 1.05 Å cutoff | Number of clusters at 1.60 Å cutoff |
| --- | --- | --- | --- |
| wt | 77 | 32 | 3 |
| **R175H** | 143 | 69 | 10 |
| **Y220C** | 108 | 53 | 3 |
| **G245S** | 126 | 57 | 4 |
| **R248Q** | 113 | 62 | 8 |
| **R249S** | 119 | 54 | 5 |
| **R273H** | 92 | 52 | 3 |
| **R282W** | 136 | 65 | 7 |
| *R273H_S240R* | 83 | 42 | 3 |
| *R273H_N263V* | 73 | 32 | 2 |
| *R273H_N200Q_D208T* | 89 | 44 | 2 |
| *R273H_N235K_N239Y* | 84 | 43 | 4 |
| *G245S_N239Y* | 54 | 27 | 2 |
| *G245S_T123P* | 135 | 67 | 7 |
| *Y220C_A138G* | 95 | 46 | 3 |
| *Y220C_L137R* | 105 | 52 | 4 |
| R273H_N239S | 96 | 53 | 4 |
| R273H_R282S | 106 | 56 | 5 |
| R273H_L114G | 114 | 57 | 4 |
| G245S_E286D | 147 | 77 | 5 |
| Y220C_L114G | 187 | 95 | 10 |
| N239Y | 69 | 33 | 3 |
| M133L_V203A_N239Y_N268D  (first 30 ns of MD simulation) | 96 | 46 | 3 |
| M133L_V203A_N239Y_N268D  (second 30 ns of MD simulation) | 52 | 22 | 1 |

Cancer mutants are typed in bold letters, rescue mutants are italicized, and non-rescue mutants are underlined.
