# Supplementary material for: Ensemble-Based Computational Approach Discriminates Functional Activity of p53 Cancer and Rescue Mutants
Source: PLoS Comput Biol. 2011 Oct 20;7(10):e1002238. doi: 10.1371/journal.pcbi.1002238 (PMC3197647; doi:10.1371/journal.pcbi.1002238)
Supplement: Table S2 — The root-mean-square-fluctuations for p53 mutants in MD trajectories. (DOC) [file pcbi.1002238.s004.doc]

**Table S2. The root-mean-square-fluctuations for p53 mutants in MD trajectories.**

|  | RMSF of all Cα atoms |
| --- | --- |
| wt | 0.879 |
| **R175H** | 1.139 |
| **Y220C** | 0.941 |
| **G245S** | 1.033 |
| **R248Q** | 1.057 |
| **R249S** | 0.926 |
| **R273H** | 0.913 |
| **R282W** | 1.036 |
| *R273H_S240R* | 0.893 |
| *R273H_N263V* | 0.858 |
| *R273H_N200Q_D208T* | 0.847 |
| *R273H_N235K_N239Y* | 0.847 |
| *G245S_N239Y* | 0.839 |
| *G245S_T123P* | 1.000 |
| *Y220C_A138G* | 0.896 |
| *Y220C_L137R* | 0.954 |
| R273H_N239S | 0.893 |
| R273H_R282S | 1.004 |
| R273H_L114G | 0.890 |
| G245S_E286D | 1.070 |
| Y220C_L114G | 1.130 |
| N239Y | 0.887 |
| M133L_V203A_N239Y_N268D  (first 30 ns of MD simulation) | 0.935 |
| M133L_V203A_N239Y_N268D  (second 30 ns of MD simulation) | 0.737 |

Cancer mutants are typed in bold letters, rescue mutants are italicized, and non-rescue mutants are underlined.
