# Supplementary material for: Ensemble-Based Computational Approach Discriminates Functional Activity of p53 Cancer and Rescue Mutants
Source: PLoS Comput Biol. 2011 Oct 20;7(10):e1002238. doi: 10.1371/journal.pcbi.1002238 (PMC3197647; doi:10.1371/journal.pcbi.1002238)
Supplement: Table S3 — The number of clusters for p53 mutants in MD trajectories using single-linkage algorithm and Jarvis-Patrick algorithm. (DOC) [file pcbi.1002238.s005.doc]

**Table S3. The number of clusters for p53 mutants in MD trajectories using single-linkage algorithm and Jarvis-Patrick algorithm.**

|  | Single-linkage | Jarvis-Patrick |
| --- | --- | --- |
| wt | 784 | 60 |
| **R175H** | 1424 | 368 |
| **Y220C** | 1096 | 118 |
| **G245S** | 1458 | 386 |
| **R248Q** | 1284 | 202 |
| **R249S** | 1460 | 302 |
| **R273H** | 1195 | 122 |
| **R282W** | 1437 | 375 |
| *R273H_S240R* | 1041 | 76 |
| *R273H_N263V* | 916 | 87 |
| *R273H_N200Q_D208T* | 1166 | 145 |
| *R273H_N235K_N239Y* | 1051 | 93 |
| *G245S_N239Y* | 693 | 36 |
| *G245S_T123P* | 1453 | 359 |
| *Y220C_A138G* | 1181 | 187 |
| *Y220C_L137R* | 1221 | 197 |
| R273H_N239S | 1161 | 169 |
| R273H_R282S | 1265 | 259 |
| R273H_L114G | 1379 | 278 |
| G245S_E286D | 1498 | 472 |
| Y220C_L114G | 1860 | 913 |
| N239Y | 973 | 75 |
| M133L_V203A_N239Y_N268D  (first 30 ns of MD simulation) | 1105 | 115 |
| M133L_V203A_N239Y_N268D  (second 30 ns of MD simulation) | 864 | 32 |

Cancer mutants are typed in bold letters, rescue mutants are italicized, and non-rescue mutants are underlined.
